# Acute Uremic Encephalopathy in an Adult Patient Due to Urinary Retention After Spinal Cord Stimulation: A Case Report and Literature Review

**DOI:** 10.7759/cureus.101924

**Published:** 2026-01-20

**Authors:** Georgi Krasimirov Georgiev, Todor Shamov, Tihomir Eftimov

**Affiliations:** 1 Department of Neurosurgery, St. Anna University Hospital, Sofia, BGR

**Keywords:** complication, encephalopathy, retention, spinal cord stimulator, uremia

## Abstract

Spinal cord stimulation (SCS), following the gate control theory of pain, has progressively evolved into an established neuromodulation technique for the treatment of refractory neuropathic pain. Although SCS is generally regarded as a safe procedure, complications are rare, with the majority related to hardware malfunction or minor biological events. Severe neurological or systemic complications are quite rare, while autonomic disturbances such as urinary retention or altered consciousness are considered exceptionally uncommon. We present a clinical case of a 59-year-old patient with urinary disturbances (retention), leading to clinical representation of uremic encephalopathy week after implantation of SCS. In parallel, a structured literature review was conducted using PubMed/MEDLINE and additional medical databases, focusing on SCS-related neurological and autonomic complications. Our report is the second to report and highlight a rare but reversible complication of SCS. Early recognition, prompt deactivation of the stimulation system, and careful reprogramming may lead to complete clinical recovery. While SCS remains an effective therapy for refractory neuropathic pain, awareness of atypical neurological and autonomic adverse effects is essential for safe clinical practice.

## Introduction

Spinal cord stimulation (SCS) has become an established and widely accepted neuromodulation technique for the management of chronic, refractory neuropathic pain, increasing pain reduction, functional improvement, and quality of life enhancement in carefully selected patients [1]. Since its first clinical application in 1967 [2], following the gate control theory of pain, SCS has undergone significant technological and conceptual evolution, expanding its indications beyond neuropathic pain to include ischemic pain syndromes, such as refractory angina pectoris and selected cases of coronary artery disease [3-5].

Although SCS is generally safe and effective, overall complication rates of up to 40% [1] have been reported, most of which are minor and manageable. Severe neurological and autonomic complications remain exceptionally rare, with only one comparable case described in the literature to date [6]. The present report adds an additional case, contributing valuable evidence to this extremely limited and potentially reversible complication profile.

## Case presentation

A 59-year-old male patient with a history of severe thoracolumbar spinal trauma presented with chronic, pharmacoresistant neuropathic pain. The initial injury resulted in a T12 burst fracture with spinal cord contusion, which was surgically stabilized by posterior transpedicular fixation (Th11-L1). Following neurological stabilization, the patient developed persistent central neuropathic pain, predominantly affecting the gluteal region and both lower extremities, with distal radiation to the feet.

The pain was described as burning and dysesthetic, associated with marked hyperalgesia and allodynia, and significantly impaired mobility. Neurological examination demonstrated distal flaccid paraparesis, reduced motor strength in the lower extremities, absent deep tendon reflexes, and sensory disturbances. Neurophysiological studies confirmed severe bilateral lumbosacral root damage, consistent with post-traumatic epiconus syndrome. MRI revealed chronic post-traumatic myelopathic changes without surgically correctable compression.

Despite prolonged and comprehensive conservative treatment, including anticonvulsants, antidepressants, opioids, ketamine infusions, sympathetic nerve blocks, and physiotherapy, the patient experienced insufficient and transient pain relief. Given the diagnosis of refractory central neuropathic pain, absence of further surgical options, and significant functional impairment, the patient was discussed at a multidisciplinary board, where SCS was recommended as the most appropriate therapeutic strategy.

Intervention and outcome

**Figure 1:**
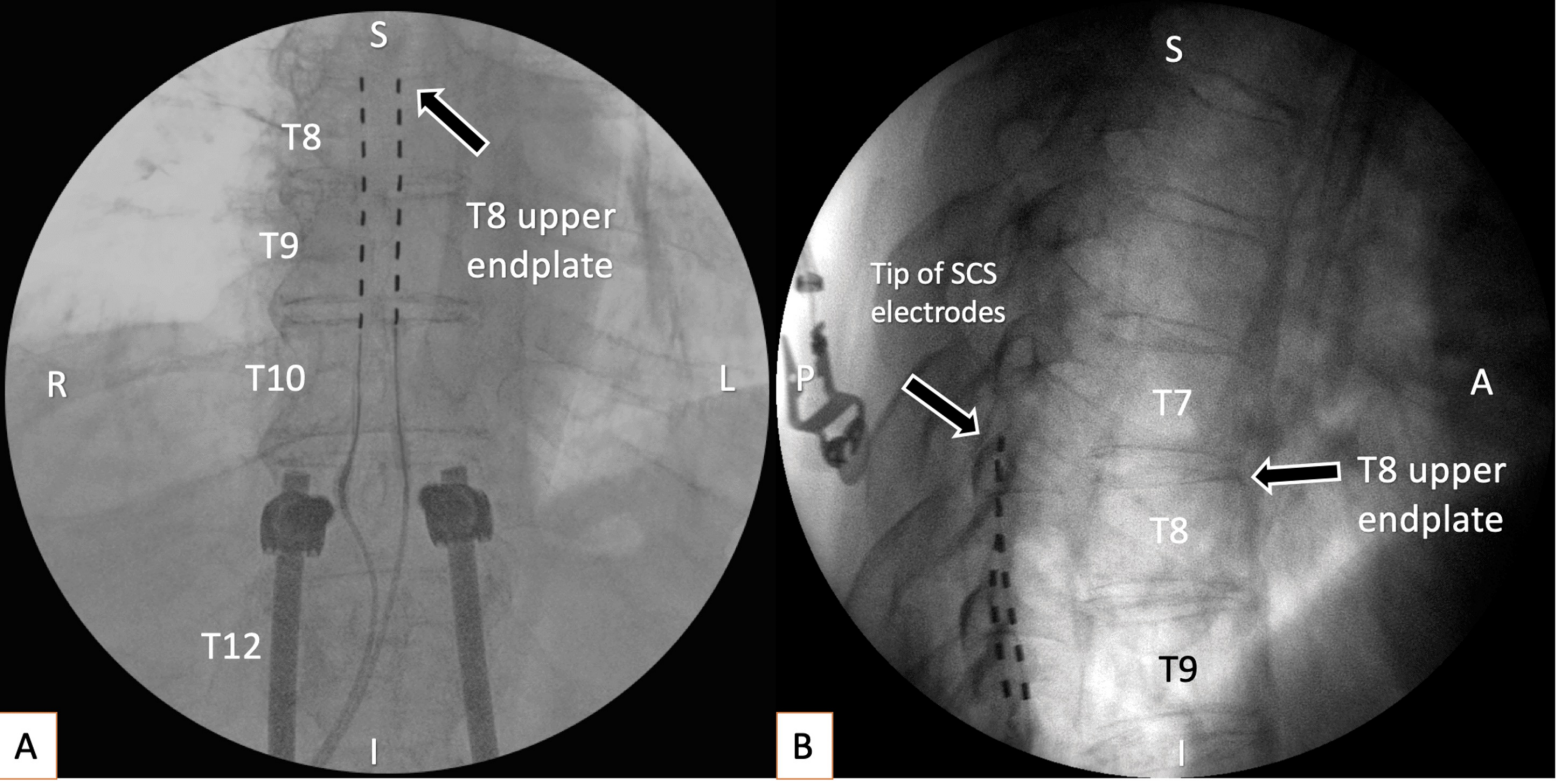
Anterior-posterior intraoperative fluoroscopy showed final positioning of electrodes at the rostral endplate of Th8 (shown with a black arrow); (B) lateral view intraoperative fluoroscopy (the upper parts of electrodes are covering each other). Both figures represent accurate placement of electrodes dorsal and symmetrical to midline A: anterior; I: inferior; L: left; P: posterior; R: right; S: superior; T8: 8th thoracic vertebra; T9: 9th thoracic vertebra; T10: 10th thoracic vertebra; T12: 12th thoracic vertebra

The patient underwent implantation of the Medtronic lntellis™ spinal cord stimulation system; the tips of the electrodes were placed at level Th 8 (Figure 1) via open surgical exposure. This exposure was preferred based on the risk of postoperative adhesions and possibility of dural puncture. Placement of the electrodes was based on optimal pain reduction and functional improvement of the patient with 1.4 V, 60Hz, 210 millisecond inter-pulse interval parameters. Permanent implantation of the stimulator was performed after two days. He was discharged in good general condition and with significant relief of the pain (Figure 1).

**Figure 2:**
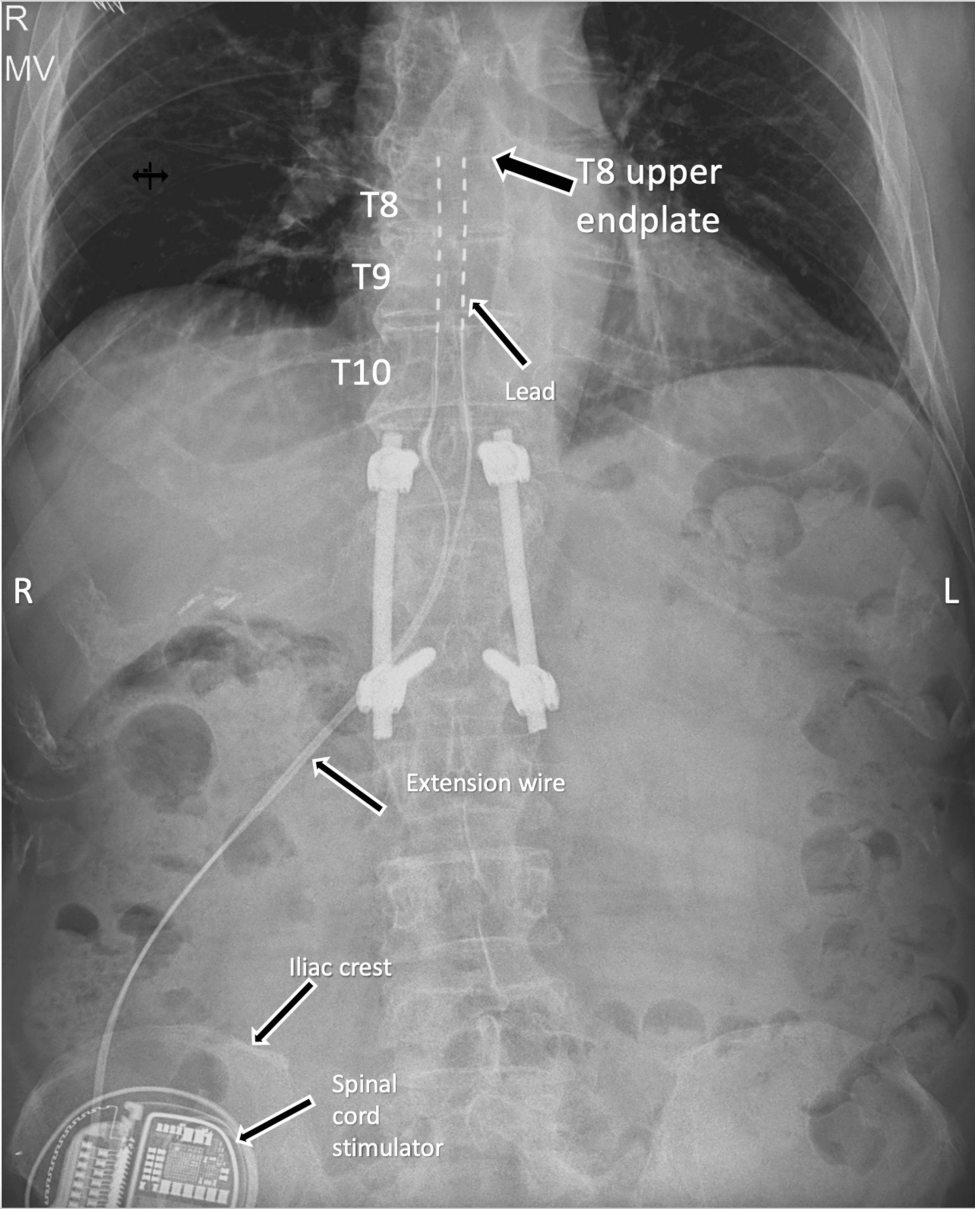
Anterior-posterior X-ray in admission to emergency room covering electrodes and programmer with no hardware migration, disconnection or lead fracture. Black arrows showing positioning of leads, extension wire, and spinal cord stimulator) T8: 8th thoracic vertebra; T9: 9th thoracic vertebra; T10: 10th thoracic vertebra; L: left; R: right

However, approximately one week after discharge, the patient was readmitted due to acute quantitative and qualitative disturbances of consciousness, accompanied by choreiform and dystonic movements. On presentation, the Glasgow Coma Scale score was approximately 8-9 points. Clinical evaluation (blood samples and ultrasound of abdominal organs) revealed globus vesicalis with acute urinary retention (approximately 2700 ml) and hydronephrosis, with laboratory evidence of secondary renal impairment, including elevated serum creatinine, urea, and uric acid levels, consistent with postrenal azotemia (normal blood count and hepatic enzymes, without granulocytosis, slightly elevated CRP 31.1 (reference range: 0-5) and urea 36.99 mmol/L (normal range: 2.5-6.7 mmol/L) and serum creatinine 1433.0 µmol/L; normal range: 65-120 µmol/L). A control head CT scan revealed no abnormalities and AP X-ray of the chest showed no changes in positioning of the electrodes or breakdown (Figure 2).

**Figure 3:**
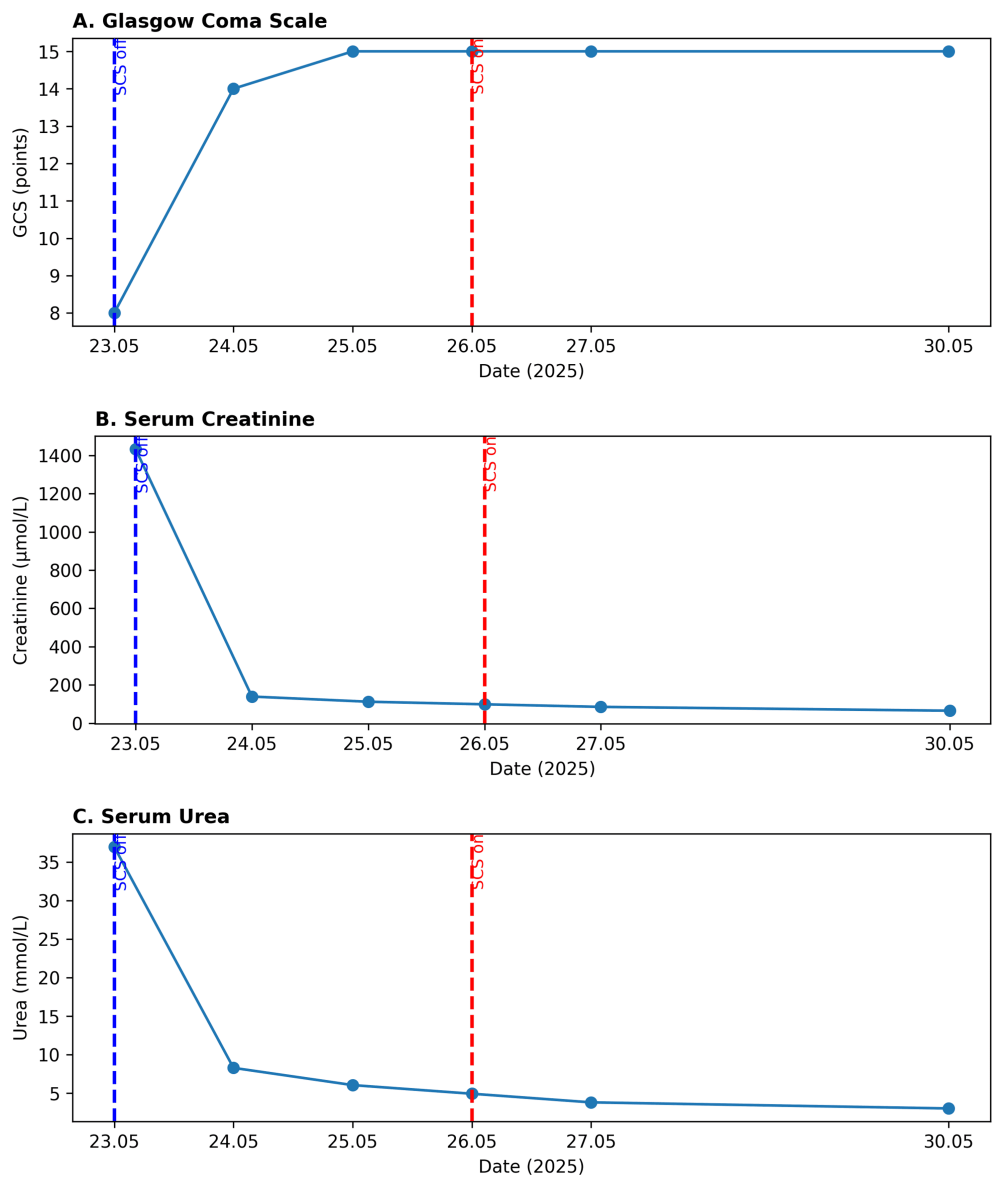
(A) Glasgow Coma Scale (GCS) dynamics with gradual improvement from 8 to 15 points between 23 and 25 May 2025. (B and C) Serum creatinine and urea levels, respectively, demonstrating rapid biochemical recovery following bladder decompression and spinal cord stimulator deactivation. The blue dashed line indicates SCS deactivation, while the red dashed line marks SCS reactivation. SCS: Spinal cord stimulation

The patient was transferred to the intensive care unit, where bladder catheterization and dialysis were performed. The spinal cord stimulator was immediately deactivated. Following device deactivation, a complete and rapid resolution of neurological symptoms was observed, along with normalization of renal function parameters (Figure 3). Subsequently, the stimulation parameters were carefully reprogrammed (0.8 V, 60Hz, 210 millisecond inter-pulse interval), allowing reactivation of the system without recurrence of neurological or autonomic symptoms, while maintaining satisfactory pain control. Four months of follow-up with pelvic ultrasound showed no residue of urine in the bladder after micturition. In addition, good control of the pain and better quality of life were achieved.

## Discussion

With expanding indications and broader clinical application, the spectrum and frequency of reported complications have been increasingly well characterized. Overall complication rates associated with SCS implantation have been reported to reach up to 40% and are generally classified into hardware-related, biological, and programming-related complications [1].

Hardware-related complications

The most frequently reported hardware-related complication is lead migration, with an incidence ranging from 2% to 27%, and a mean reported rate of approximately 15.49% [1]. Lead migration may compromise stimulation coverage and clinical efficacy. The second most common hardware complication is lead fracture and disconnection (0-10.2%; mean 6.37%) [1]. Other hardware-related issues include battery malfunction or generator displacement, reported in approximately 1.7% to 10% of patients [7,8].

Biological complications

Among biological complications, pain at the implant site is reported with a wide incidence range from 0.9% to 12% [1]. Infectious complications represent a significant concern, with reported rates between 2.5% and 10%, potentially necessitating device removal [1]. Skin erosion overlying the implanted components has been described in 0.2% cases [7].

Hematoma formation, particularly epidural hematoma, is a rare complication reported in approximately 0.3% of cases. Similarly, dural puncture or inadvertent dural perforation during lead or catheter insertion has been documented in roughly 0.3% of patients [7]. Despite its low incidence, dural puncture may result in cerebrospinal fluid leakage and subsequent intracranial hypotension, which can manifest clinically with postural headache, nausea, altered consciousness, or neurological deterioration. In selected cases, conservative management is insufficient, and surgical intervention may be required to definitively address persistent cerebrospinal fluid leakage and its intracranial sequelae [1]. Importantly, permanent neurological injury remains exceedingly rare, with an estimated incidence ranging between 0.03% and 0.25% of all implanted patients [7,9].

Programming-related complications

The third category of complications involves programming-related disturbances, most commonly related to sensory stimulation abnormalities. The most frequent of these is loss of paresthesia, reported in approximately 5% to 15% of patients. This phenomenon is most often attributed to lead migration, lead fracture, epidural fibrosis, or progression of the underlying disease [7].

In contrast, painful or dysesthetic paresthesia occurs less frequently, with a reported incidence of approximately 2% to 7% [7]. This complication is typically associated with inappropriate stimulation parameters, suboptimal electrode positioning, unintended stimulation of off-target neural structures, or altered excitability of the spinal cord dorsal columns. In most cases, painful paresthesia is reversible with careful reprogramming of stimulation parameters [1].

Bladder disturbance is a rare complication in SCS with a frequency of 0.3-0.8% of all SCS, but only few cases have been reported with urinary retention until now [6,10,11]. Possible cause is disturbance, based on local interference in the neural network represented in the superficial dorsal horns, intermediolateral region, and posterior commissure [10]. Additionally, activation of spinal segments T10-L2 may induce urinary retention through stimulation of the hypogastric plexus, which provides sympathetic regulation of the pelvic organs and bladder. Sympathetic activation leads to relaxation of the bladder detrusor muscle and contraction of the urethral sphincter, which, during SCS, may override parasympathetic pelvic splanchnic input and result in pathological urinary retention [6]. Sometimes SCS on the T10 spinal segment can stimulate both detrusor and external bladder sphincter muscles due to its anatomical proximity, resulting in functional urinary retention [12]. Moreover, SCS can inhibit or modulate afferent and efferent signaling between the spinal cord and the pontine and cortical micturition centers, thereby altering their regulatory output. This dysregulation may mimic urinary dysfunction typically observed in localized spinal cord injury, while preserving somatic sensory and motor function. This is well represented in cases in SCS in the cervical region, causing urinary disturbances [10].

Our case is a rare presentation of programming-related complication, which led to biological (autonomic) uremic encephalopathy due to severe urinary retention, which may be used as an example in refining the SCS complication list as a distinct or subtype of the existing one.

## Conclusions

SCS remains a valuable therapeutic option for pharmacoresistant central neuropathic pain. Urinary retention with subsequent uremic encephalopathy represents an exceptionally rare but potentially life-threatening complication of SCS. This condition should be promptly considered and actively evaluated in patients presenting with acute altered consciousness following SCS, including early bedside assessment with abdominal and bladder ultrasonography, as well as mandatory measurement of serum creatinine and urea levels. When indicated, immediate urinary catheterization combined with supportive measures aimed at reducing uremia, including appropriate pharmacological management, constitutes the first-line treatment. Importantly, this condition is rapidly and fully reversible through timely deactivation and appropriate reprogramming of the spinal cord stimulator, allowing preservation of therapeutic benefit while eliminating risks to the patient’s health and survival.

## References

[1] Eldabe S, Kumar K, Buchser E, Taylor RS (2010). An analysis of the components of pain, function, and health-related quality of life in patients with failed back surgery syndrome treated with spinal cord stimulation or conventional medical management. Neuromodulation.

[2] Shealy CN, Mortimer JT, Reswick JB (1967). Electrical inhibition of pain by stimulation of the dorsal columns: preliminary clinical report. Anesth Analg.

[3] Cruccu G, Aziz TZ, Garcia-Larrea L (2007). EFNS guidelines on neurostimulation therapy for neuropathic pain. Eur J Neurol.

[4] Ubbink DT, Vermeulen H (2006). Spinal cord stimulation for critical leg ischemia: a review of effectiveness and optimal patient selection. J Pain Symptom Manage.

[5] Taylor RS, De Vries J, Buchser E, Dejongste MJ (2009). Spinal cord stimulation in the treatment of refractory angina: systematic review and meta-analysis of randomised controlled trials. BMC Cardiovasc Disord.

[6] La Grua M, Michelagnoli G (2010). Rare adverse effect of spinal cord stimulation: micturition inhibition. Clin J Pain.

[7] Cameron T (2004). Safety and efficacy of spinal cord stimulation for the treatment of chronic pain: a 20-year literature review. J Neurosurg.

[8] Turner JA, Loeser JD, Deyo RA, Sanders SB (2004). Spinal cord stimulation for patients with failed back surgery syndrome or complex regional pain syndrome: a systematic review of effectiveness and complications. Pain.

[9] Levy R, Henderson J, Slavin K, Simpson BA, Barolat G, Shipley J, North R (2011). Incidence and avoidance of neurologic complications with paddle type spinal cord stimulation leads. Neuromodulation.

[10] Smeijers S, Kho KH, De Vlieger J, Van Hoylandt A, Nuttin B, Theys T (2022). Spinal cord stimulation and urinary dysfunction. Pain Med.

[11] Loubser PG (1997). Adverse effects of epidural spinal cord stimulation on bladder function in a patient with chronic spinal cord injury pain. J Pain Symptom Manage.

[12] Nashold BS Jr, Friedman H, Boyarsky SJ (1971). Electrical activation of micturition by spinal cord stimulation. Surg Res.

